# Plant growth-promoting properties of *Streptomyces* spp. isolates and their impact on mung bean plantlets’ rhizosphere microbiome

**DOI:** 10.3389/fmicb.2022.967415

**Published:** 2022-08-25

**Authors:** Napawit Nonthakaew, Watanalai Panbangred, Wisuwat Songnuan, Bungonsiri Intra

**Affiliations:** ^1^Department of Biotechnology, Faculty of Science, Mahidol University, Bangkok, Thailand; ^2^Osaka Collaborative Research Center for Bioscience and Biotechnology, Mahidol University-Osaka, Bangkok, Thailand; ^3^Research, Innovation, and Partnerships Office (Office of the President), King Mongkut’s University of Technology Thonburi, Bangkok, Thailand; ^4^Department of Plant Science, Faculty of Science, Mahidol University, Bangkok, Thailand

**Keywords:** *Streptomyces*, plant growth-promoting rhizobacteria (PGPR), plant-beneficial genes, *Phytophthora palmivora*, microbiome, *Streptomyces* exploration, biological control

## Abstract

*Phytophthora* is an important, highly destructive pathogen of many plants, which causes considerable crop loss, especially durians in Thailand. In this study, we selectively isolated *Streptomyces* from the rhizosphere soil with a potent anti-oomycete activity against *Phytophthora palmivora* CbP03. Two strains (SNN087 and SNN289) demonstrated exceptional plant growth-promoting properties in pot experiment. Both strains promoted mung bean (*Vigna radiate*) growth effectively in both sterile and non-sterile soils. Metagenomic analysis revealed that *Streptomyces* sp. SNN289 may modify the rhizosphere microbial communities, especially promoting microbes beneficial for plant growth. The relative abundance of bacterial genera *Bacillus*, *Sphingomonas*, *Arthrobacter*, and *Pseudarthrobacter*, and fungal genera *Coprinellus* and *Chaetomium* were noticeably increased, whereas a genus *Fusarium* was slightly reduced. Interestingly, *Streptomyces* sp. SNN289 exhibited an exploratory growth, which allows it to survive in a highly competitive environment. Based on whole genome sequence analysis combined with an ANI and dDDH values, this strain should be classifiable as a new species. Functional annotation was also used to characterize plant-beneficial genes in SNN087 and SNN289 genomes for production of siderophores, 3-indole acetic acid (IAA), ammonia, and solubilized phosphate. AntiSMASH genome analysis and preliminary annotation revealed biosynthetic gene clusters with possible secondary metabolites. These findings emphasize the potential for application of strain SNN289 as a bioinoculant for sustainable agricultural practice.

## Graphical Abstract

“Created with BioRender.com”. 
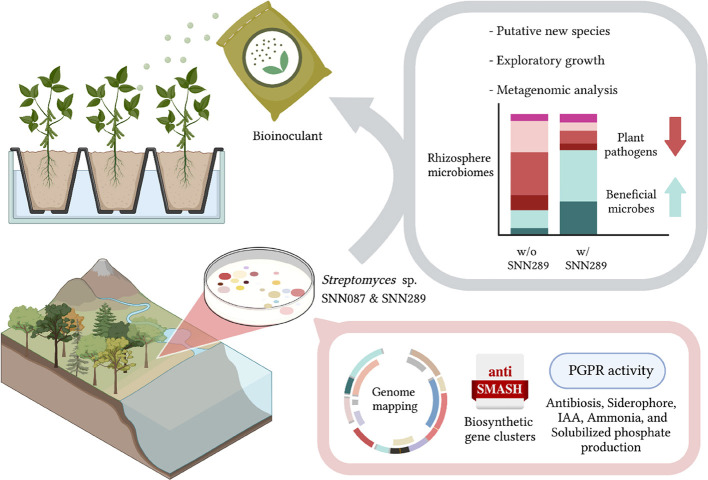

## Introduction

The need for sustained high quality of food while increasing yields to feed a growing world population remains one of the most urgent challenges for the agricultural sector (Alexandratos and Bruinsma, 2012). Crop production in conventional practice uses mainly synthetic pesticides and fertilizers to achieve these goals, resulting in serious environmental issues such as soil contamination and deterioration, water pollution, biodiversity loss, and adverse health effects in humans (Rivera et al., 2017). Plant growth-promoting rhizobacteria (PGPR) have gained attention as an alternative approach to maintaining soil fertility and promoting crop growth in a sustainable manner, due to their innate biological processes that promote plant health, including phytohormone production, phosphate solubilization, mineralization, nitrogen fixation, degrading enzymes, etc. (Chandran et al., 2021).

Actinobacteria are Gram-positive bacteria whose genomes contain a high proportion of guanine and cytosine nucleotides (Ventura et al., 2007). In recent years, Actinobacteria along with the Firmicutes have been appreciated for the pivotal role they play in suppressing plant pathogens (Lee et al., 2021; Solís-García et al., 2021). *Streptomyces*, the largest genus of the phylum Actinobacteria, are spore-forming bacteria that are well-characterized as a genus, owing to their role as a rich source of antibiotics used in the medical and agricultural sectors (Bibb, 2013). *Streptomyces* are saprophytes that degrade complex organic substances, such as chitin and cellulose, using their lytic enzymes (Chater, 2016). Those same characteristics enable them to survive in unfavorable conditions and in highly competitive environments. Similar to other PGPR, *Streptomyces* can promote plant growth and plant uptake of nutrients through their metabolites or specialized bioactivities, including auxin and ammonia production, iron chelation by bacterial siderophores, and phosphate solubilization (Olanrewaju et al., 2017; Suárez-Moreno et al., 2019). Many studies to date have extensively described their effects on plant growth (Anwar et al., 2016; Suárez-Moreno et al., 2019; Worsley et al., 2020), however, only a few reports have examined their effects on microbial community dynamics.

*Phytophthora palmivora* is a broad host-range oomycete pathogen commonly found in tropical and sub-tropical regions. This species causes destructive diseases in a wide range of economic crops, including trunk canker, root rot, and fruit rot of durian (Tongon et al., 2018, black pod and stem canker of cocoa (Awuah and Frimpong, 2002) and jackfruit (Tri et al., 2015), and patch canker, black stripe, green pod rot of rubber (Sdoodee, 2004). In this study, *Streptomyces* spp. isolated from the rhizosphere soil of durian (*Durio zibethinus*) were selected based on strong inhibitory activity against *P. palmivora* before they were evaluated for plant growth promoting traits and to test benefits to mung bean (*Vigna radiate*, a representative of dicotyledons), based on its putative beneficial effects on economically important crops in Thailand, such as durians. We identified their effect on the soil microbial community, and report genome-wide analyses of proteins responsible for plant growth promotion as well as biosynthetic gene clusters (BGCs) for secondary metabolite production.

## Materials and methods

### Isolation of plant growth-promoting *Streptomyces* isolates

Soil samples were collected from five rhizosphere soils of star gooseberry (*Phyllanthus acidus*) in Bangkok, and longkong (*Lansium domesticum*) and durian (*Durio zibethinus*) in Chanthaburi Province, Thailand. Soil samples were dried at room temperature for 1 week. One gram of soil was suspended in 9 ml of sterile normal saline solution and serially diluted 10-fold to obtain dilutions of 10^–3^, 10^–4^ and 10^–5^. Subsequently, 0.1 ml of each dilution was spread on Pridham’s agar [g/l: glucose, 10.0; starch, 10.0; (NH_4_)_2_SO_4_, 2.0; CaCO_3_, 2.0; K_2_HPO_4_, 1.0; MgSO_4_, 1.0; NaCl, 1.0; agar, 12.0] and water-proline agar (g/l of tap water: proline, 10.0; agar, 12.0). To selectively isolate actinobacteria, both agars were supplemented with 25 μg/ml nalidixic acid and 50 μg/ml cycloheximide, which are used to inhibit gram-negative bacteria and fungi, respectively. The plates were incubated at 30°C for 3–7 days. The colonies with filamentous features were selected and streaked on Pridham’s or water-proline agars.

### Screening of antimicrobial activity

Rhizosphere isolates were cultured on 301 agar (g/l: calcium carbonate, 4.0; glucose, 1.0; starch, 24.0; meat extract, 3.0; peptone, 3.0; yeast extract, 5.0; agar, 12.0) at 30°C for 7 days. *Phytophthora palmivora* CbP03, a plant pathogen resistant to metalaxyl fungicide, was grown on in parallel 301 agar for 7 days in a similar manner as the isolate. A sterile Cork borer was used to cut the oomycete and the isolated rhizosphere isolate strains (8 mm in diameter). The agar block containing the oomycete was placed on a 301 agar plate, while the agar block of the isolated strains was placed 2 cm away on the same plate. The plates were incubated at 30°C for 7 days, after which the length of the inhibition zone between the fungal and bacterial cores was measured.

### Identification of *Streptomyces*-like isolates using *16S rRNA* gene analysis

Four bacterial isolates were selected for their strong inhibitory activity against *P. palmivora* CbP03 for further characterization by *16S rRNA* gene sequencing. The bacterial biomass was prepared by growing in liquid 301 medium at 30°C, 200 rpm for 3 days. The bacterial cells were harvested by centrifugation at 8,000 rpm for 10 min and resuspended in Type 1 water. Next, the cells were resuspended in 500 μl of 1X Tris-EDTA (TE) buffer (1 l; 10 ml of 10 mM Tris–HCl, pH 8; 10 ml of 1 mM Na_2_EDTA, pH 8; 980 ml Type 1 water) before homogenization using a tissue grinder. PCR amplification of the *16S rRNA* gene was carried out using iStarTaq polymerase and 2X PCR Master mix solution and Universal primers 11F (5′ AGTTTGATCATGGCTCAG 3′) and 1540R (5′ AAGGAGGTGATCCAGCCGCA 3′) on a thermal cycler (Perkin Elmer GeneAmp PCR System 2400 Thermal Cycler). PCR fragments of the expected length (∼1,500 bp) was confirmed by agarose gel electrophoresis (1% w/v), and the products were gel purified using PureLink Quick Gel Extraction and PCR Purification Combo Kit (Invitrogen), according to the manufacturer’ instructions. The purified PCR products were commercially sequenced at Macrogen, Korea. To clarify phylogenetic relationships to related species, the resultant sequences were compared to similar sequences in the EzBioCloud database.^1^ Phylogenetic trees based on a neighbor-joining approach were constructed and analyzed using MEGA 7 software (Kumar et al., 2016). Bootstrap analysis with 1,000 re-sampled datasets was used to evaluate the resultant tree topology. Strain SNN289 was independently identified based on whole genome sequence analysis. An ANI value was calculated in the EzBioClound, while a digital DNA-DNA hybridization (dDDH) was calculated using the Genome-to-Genome Distance Calculator (GGDC 3.0) combined with the BLAST function (Meier-Kolthoff et al., 2022).

### *In vitro* determination of plant growth-promoting activities

#### Determination of phosphate solubilization

The selected isolates were inoculated in 5 ml of ISP-2 broth and grown with shaking at 200 rpm, 30°C for 3 days. The cultures were then inoculated into 100 ml of NBRIP broth [glucose, 10 g: Ca_3_(PO_4_)_2_, 5 g; MgCl_2_•6H_2_O, 5 g; MgSO_4_•7H_2_O, 0.25 g; KCl, 0.2 g and (NH_4_)_2_SO_4_, 0.1 g] (Nautiyal, 1999) and shaken at 170 rpm, 30°C for 5 days. After cultivation, the soluble phosphate was determined as described in Murphy and Riley (1962).

#### Determination of indole-3-acetic acid and 3-indole acetic acid-like substances

The selected strains were cultivated in 5 ml of tryptic soy broth (TSB) (g/l: glucose, 2.5; tryptone, 17.0; soy, 3.0; NaCl, 5.0; K_2_HPO_4_, 2.5) with and without 1 mg/ml of L-tryptophan. The tubes were shaken at 200 rpm, 30°C for 7 days. Cultures were centrifuged 12,000 rpm for 10 min and 1 ml of the supernatant was transferred to a microfuge tube. Two milliliters of Salkowski’s reagent (0.5 M FeCl_3_ in 35% perchloric acid) was added to the test supernatant and the mixture was incubated at room temperature for 30 min in the dark (Gordon and Weber, 1951). The appearance of a pink color indicates the presence of indole compounds in the supernatant. Absorbance of the supernatant was measured using a spectrophotometer (wavelength = 530 nm) on 200 μl of each sample, which permitted calculation of the concentration of IAA-like substances.

To quantify the amount of IAA using high- performance liquid chromatography (HPLC), test strains were cultivated in 5 ml of ISP-2 medium for 3 days. Then each was inoculated into 100 ml of TSB medium. The flasks were shaken at 200 rpm, 30°C for 7 days. After a 7-day fermentation, the culture was centrifuged at 8,000 rpm for 10 min to collect the cell pellet, and the supernatant was transferred to a new vessel, where the pH of the supernatant was adjusted to pH 2.5. One hundred milliliters of ethyl acetate were poured into a separatory funnel to extract IAA. The organic phase was collected and evaporated to obtain crude extract. A C18 reverse phase column (Cadenza CD-C18 reverse phase symmetry column; 4.6 mm × 210 mm, 3.5 μm pore size) run on an Agilent HP1100 system (Hewlett-Packard) coupled with a photodiode array UV-VIS detector (190–600 nm) was used for IAA quantitative analysis. The temperature of the column was maintained at 40°C. Mobile phase of 20% CH_3_CN/0.1% HCOOH flowed at a rate of 1 ml/min, and the detector was set to 280 nm for 30 min (Carreño-Lopez et al., 2000).

#### Ammonia production

Ammonia production of the test strains was tested in peptone water (g/l: Peptone 10.0; sodium chloride 5.0). Freshly grown cultures were inoculated into 5 ml of peptone water and cultivated at 30°C, 200 rpm for 7 days. Nessler’s reagent (0.5 ml) (Merck, Germany) was added into each bacterial suspension. Orange precipitate was noted as a positive result for ammonia production.

#### Siderophore production

Bacterial isolates were examined for siderophore production on a chrome azurol sulfate (CAS) agar, as described by Schwyn and Neilands (1987). The *Streptomyces* isolates were inoculated onto CAS agar and grown at 30°C for 3–7 days. Development of a yellow-orange halo around the colonies was noted as positive for siderophore production.

#### Extracellular degrading enzymes production

Test strains were streaked onto Bushnell Haas medium (BHM) agar plates amended with CMC sodium salt as the sole carbon source, to assay cellulose production, or xylan (from birchwood) as the sole carbon source, to assay xylanase production (g/l: CMC/xylan, 10; MgSO_4_•7H_2_O, 0.2; K_2_HPO_4_, 1; KH_2_PO_4_, 1; NH_4_NO_3_, 1.0; FeCl_3_•6H_2_O, 0.05; CaCl_2_, 0.02; agar, 12). After incubation at 30°C for 7 days, the plates were visualized using Gram’s iodine (2.0 g KI and 1.0 g iodine in 300 ml distilled water) to observe the hydrolytic activities of isolated strains.

For chitin degradation, the isolates were streaked onto colloidal chitin agar (g/l: colloidal chitin, 10; Na_2_HPO_4_, 6; KH_2_PO_4_, 3; NH_4_Cl, 1; NaCl, 0.5; yeast extract, 0.05; agar, 15) (Saima and Roohi, 2013). The appearance of clear zone around the colonies was noted as an indication of chitinase production.

#### Evaluation of growth under variable salinity, pH, and temperature

The selected isolates were streaked onto ISP-2 agar containing varying NaCl concentrations of 0, 4, 7, 10, and 13%. The plates were incubated at 30°C for 7 days and scored for bacterial growth. To assay growth potential over a range of pH, the selected isolates were streaked on ISP-2 agar adjusted to either pH 5, 7, 9, or 11. The plates were incubated at 30°C for 7 days and scored for bacterial growth. To assay growth over a range of temperatures, the selected isolates were streaked on ISP-2 agar, and the plates were incubated at either 20, 30, 40°C of 50°C for 7 days, and scored for bacterial growth once, at the end of the incubation period.

#### Activation of exploratory growth by *Saccharomyces cerevisiae*

Unique adaptations, such as exploratory behavior, have been suggested to permit *Streptomyces* to outcompete other microbes in highly competitive environments (Jones et al., 2019). Therefore, we assayed our test strains for such exploratory behavior which cells will adopt a non-branching vegetative hyphal conformation. Cells were grown in 5 ml of TSB broth at 30°C, 200 rpm for 3 days, and on the last day, a culture of *S. cerevisiae* was grown in 5 ml of YPD broth (g/l; dextrose, 20; peptone, 20; yeast extract, 10) overnight. Three microliters of the selected bacterial culture were dropped next to 3 μl of *S. cerevisiae* on the surface of a YPD agar plate (Jones et al., 2017). Plates were then incubated at 30°C for up to 21 days.

#### Plant pot experiment

In *in vitro* plant growth-promotion assessments using Mung beans (*Vigna radiate*, a representative of dicotyledons) grown in both sterile (being autoclaved twice) and non-sterile soils, strains SNN087 and SNN289 exhibited robust phosphate-solubilizing activity with moderate IAA production. Therefore, both strains were further tested for their ability to promote plant growth in pots. For this assay, each bacterial isolate was cultivated in 50 ml of TSB medium for 3 days at 30°C, with shaking at 200 rpm (two independent colonies for each strain). Cells were centrifuged, and the pellet washed and resuspended in sterile water before being adjusted to OD_600_ = 1.00. Mung bean seeds (Raithip Brand, Thai Cereal World Company, Thailand) were surface sterilized with 5% (v/v) sodium hypochlorite for 1 min, and then washed several times with sterile water. A total of 100 μL of the remaining water from washing the seeds was spread to confirm surface sterility on nutrient agar. Mung bean seeds were then pre-germinated on sterile tissue paper in the dark for 1 day. A total of 10 mL of each inoculum was mixed with 500 g of soil. Two independent replicates of growth promotion assessment were carried out, using three conditions in each experiment: sterile water (as a control), cell suspension of strain SNN087, and cell suspension of strain SNN289. Two replicates of ten germinated seeds each were inoculated in 15 cm × 15 cm pots for each treatment at ambient conditions. After 14 days post-inoculation, root length, shoot length and fresh weight of each plant were measured. Subsequently, plants were dried at 50°C until they obtained a steady weight, and then dry weight was determined. Soil from each treatment was combined into a single rhizosphere soil sample and then maintained at −20°C for microbiome analysis.

### Microbiome analysis

#### DNA extraction and sequencing

Based on plant growth promotion results, non-sterile soil inoculated with SNN289 and control soil (non-sterile soil without inoculation) were selected for detailed analysis of changes in microbial community structure. Soils were transferred and stored in DNA/RNA Shield collection tubes during transportation to Zymo Research. The samples were processed and analyzed using the ZymoBIOMICS Service: Targeted Metagenomic Sequencing (Zymo Research, Irvine, CA, United States). ZymoBIOMICS-96 MagBead DNA Kit was used to isolate genomic DNA for sequencing. Prior to library preparation, PCR inhibitors were removed from DNA using the Zymo Research OneStepTM PCR Inhibitor Removal Kit.

The *Quick*-*16S* NGS Library Prep Kit was used to prepare DNA samples for targeted sequencing using proprietary primers selected for maximal coverage while maintaining a high level of sensitivity. The primer sets used in this project were *Quick*-*16S* Primer Set V3-V4 and ZymoBIOMICS Services ITS2 Primer Set. The sequencing library was prepared using an innovative, in-house library preparation protocol with real-time PCR monitoring to ensure prevention of PCR chimera development. The final PCR products were quantified with qPCR fluorescence readings and pooled together based on equal molarity. The pooled library was then cleaned and concentrated using the Select-a-Size DNA Clean and Concentrator. The final pooled library was cleaned using the Select-a-Size DNA Clean and Concentrator and quantified using TapeStation (Agilent Technologies, Santa Clara, CA, United States) and Qubit (Thermo Fisher Scientific, Waltham, WA, United States). As a positive control for each targeted library preparation, the ZymoBIOMICS Microbial Community DNA Standard was used. To determine the level of bioburden carried by the wet-lab process, negative controls (i.e., blank extraction control, blank library preparation control) were included. The final library was sequenced using a v3 reagent kit (600 cycles) on Illumina MiSeq. The sequencing was carried out with 10% PhiX spike-in.

#### Absolute abundance quantification

A standard curve was used to set up a quantitative real-time PCR. The standard curve was constructed using 10-fold serial dilutions of plasmid DNA containing one copy of the *16S* gene and one copy of the fungal ITS2 region. The primers used in targeted library preparation were identical to those used in targeted library preparation. For each sample, the number of gene copies in the reaction was calculated using the equation derived from the plasmid DNA standard curve. The number of gene copies per microliter in each DNA sample was calculated using the PCR input volume (2 μl).

#### Bioinformatics analysis

The Dada2 pipeline (Callahan et al., 2016) was used to infer unique amplicon sequences from raw reads. Additionally, chimeric sequences were eliminated using the Dada2 pipeline. Uclust from Qiime v.1.9.1 was used to assign taxonomy. Taxonomy was assigned using the Zymo Research Database as a reference, a *16S* database that was designed and curated internally. We used a cutoff of at least 90% similarity to a reference genome for taxonomic identification at any level. Sequence similarity below the 90% threshold was classified as “None; other.” Composition visualization and alpha-diversity analyses were performed with Qiime v.1.9.1 (Caporaso et al., 2010). Other analyses such as heatmap generation and Taxa2SV_deomposer were carried out using custom scripts.

#### Whole genome sequencing and *in silico* genome analyses for plant growth-promoting properties and predicted biosynthetic gene clusters

The draft genomes of *Streptomyces* sp. SNN087 and SNN289 were analyzed and compared using the RAST annotation server (Aziz et al., 2008) and SEED viewer (Overbeek et al., 2014) for plant-beneficial genes. A search for biosynthetic gene clusters encoding secondary metabolites was performed using antiSMASH version 6.0 (Blin et al., 2021).

### Statistical analyses

For all statistical analyses in the paper, data are presented as mean ± standard deviation. Results were analyzed by one-way analysis of variance (ANOVA) combined with the Tukey’s post-test using the SPSS statistical package (version 18.0 for Windows; SPSS Inc., Chicago, IL, United States). The level of statistical significance was considered at a *p*-value of < 0.05.

## Results

### Isolation and identification of *Streptomyces* strains with antimicrobial activity against *Phytophthora palmivora* CbP03

**FIGURE 1:**
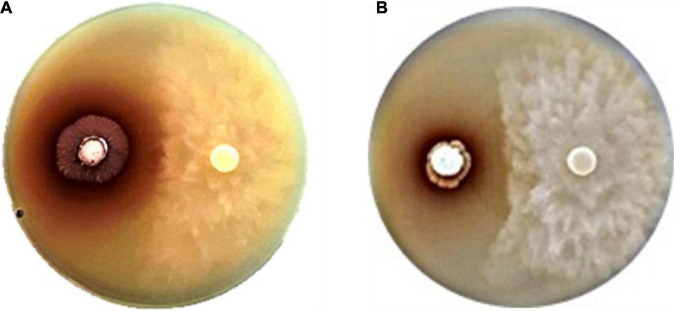
Anti-oomycete activity of strains **(A)** SNN087 **(B)** and SNN289 against *Phytophthora pamivora* CbP03 on a 301 agar.

We identified four strains exhibiting potent inhibition of *P. palmivora* CbP03: Strain SNN087, isolated from star gooseberry, SNN211, isolated from longkong, and SNN289 and SNN312, which were isolated from Monthong durian (Figure 1). *16S rRNA* sequencing analysis confirmed that all four strains were members of the genus *Streptomyces*. The closest known strains to strains SNN087, SNN211, SNN289, and SNN312 were *Streptomyces spongiae* Sp080513SC-24*^T^* (99.00% similarity), *Streptomyces spectabilis* NBRC 13424*^T^* (99.86% similarity), *Streptomyces racemochromogenes* NRRL B-5430*^T^*/*Streptomyces polychromogenes* NBRC 13072*^T^* (99.36% similarity), and *Streptomyces panaciradicis* 1MR-8*^T^* (98.65% similarity), respectively.

**FIGURE 2:**
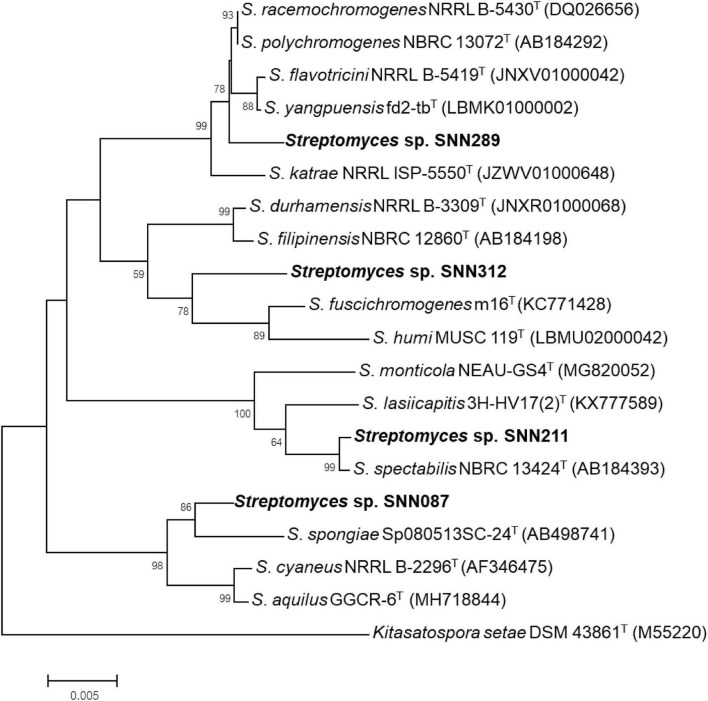
Phylogenetic tree using Neighbor-joining approach based on *16S rRNA* gene sequences of *Streptomyces* isolates. Strain *Kitasatospora setae* DSM 43861T was used as outgroup. Bootstrap values greater than 50% are displayed at branch nodes, based on 1,000 replicates and the scale bar indicates 0.005 nucleotide substitutions per site.

We constructed phylogenetic trees with the selected isolates, based on a Neighbor-joining approach (Figure 2). Several isolates showed a resemblance to the closest matches, as identified using *16S rRNA* sequencing. Strain SNN087 was placed in the *S. spongiae* clade (the closest strain) with a 90% bootstrap score, and SNN211 also fell into the closest strain (*S. spectabilis*) with a 93% bootstrap support. In contrast, strain SNN289 was classified as most closely related to *S. katrae* (98.8% similarity) with shortest evolutionary distance, and it formed a distinct clade with a bootstrap value higher than 50%. SNN312 was also placed in an adjacent branch but distinct monophyletic lineage as the clade of *S. filipinensis* (98.5% similarity), and was far away from *S. panaciradicis*, the most related strain. An average nucleotide identity (ANI) value (a measure of genome similarity) was calculated for SNN289 and closely matched species *S. katrae* NRRL ISP-5550 (Supplementary Figure 1). An ANI value of 89.10% and a dDDH value (formula 2) of 37.80% were obtained between *Streptomyces* sp. SNN289 and *S. katrae*. Since these values were below the 95% ANI threshold for same species, SNN289 was deemed to be a distinct species.

### *In vitro* plant growth-promoting activities

Two of the four initial strains were capable of solubilizing inorganic phosphate (greater than 382 μg/ml) with a remarkable reduction in the pH (Table 1). The strain most efficient in solubilizing inorganic phosphate was SNN289 (500.25 μg/ml) which also led to a significant reduction in pH to 4.68, while strain SNN211 could not improve phosphate solubilization.

**TABLE 1:** Plant growth-promoting activities by selected isolates.

Strain(SNN)	Phosphate solubilization		Auxin production			NH_3_	Siderophore	Lytic enzymes		
	Solubilized Phosphate (μg/ml)	pH	Indole compounds (μg/ml)		IAA (ng/ml)					
			No L-tryptophan	1 mg/ml L-tryptophan				Cellulase	Xylanase	Chitinase
087	382.07 ± 2.31^a^	4.54 ± 0.07^a^	43.41 ± 0.62^a^	63.93 ± 1.62^a^	905.17 ± 17.46^a^	+	+	+	+	+
211	0^b^	6.69 ± 0.04^b^	6.12 ± 0.57^b^	9.29 ± 0.11^b^	0^b^	+	+	+	+	+
289	500.25 ± 22.79^c^	4.68 ± 0.05^c^	7.64 ± 1.38 ^b^	41.03 ± 0.40^c^	115.96 ± 4.18^c^	+	+	+	+	+
312	70.46 ± 1.99^d^	6.85 ± 0.03^d^	34.17 ± 0.67^c^	40.10 ± 0.57^c^	1980.61 ± 27.73^d^	+	+	+	+	+

Results shown are the means of three values ± SD of three replicates. Different letters represent significant differences according to the one way-ANOVA combined with the Tukey’s test (*p* < 0.05). +, positive result; −, negative result.

Production of indole compounds by the selected strains were determined using colorimetric assays. These assays demonstrated that each strain was able to produce indole compounds in TSB and TSB with 1 mg/ml of tryptophan, to concentrations ranging from 6.12 to 43.42 μg/ml and 9.29–63.93 μg/ml, respectively. Strain SNN087 produced the greatest amount of indole compounds in both TSB and TSB supplemented with tryptophan, generating 43.42 and 63.93 μg/ml, respectively. This strain exhibited a noticeable increase in indole compound production in TSB supplemented with L-tryptophan, which was evident by eye in its more intense pink color. We quantified the amount of IAA produced using HPLC, and found that strains SNN087 and SNN312 produced 0.905 ± 0.017 ng/mL and 1.981 ± 0.028 ng/ml of IAA, respectively. However, IAA was not detectable in the TSB medium supernatant from SNN211 culture growth. All four isolates were found to produce siderophores, ammonia, and the enzymes cellulase, xylanase, and chitinase.

### Growth under variable salinity, pH, and temperature

The growth of each natural isolate under variable growth conditions is summarized in Table 2. All isolates grew in unmodified ISP-2. Strains SNN087 and SNN211 were able to grow in an ISP-2 modified with high salinity, up to 7% NaCl, while only strain SNN312 could only grow in ISP-2 with 0% NaCl. All four isolates could grow at all conditions within a gradient of pH from pH 5 to pH 11, and temperatures between 20°C and 40°C were also found to be optimal for the growth for all strains.

**TABLE 2:** Growth of *Streptomyces* isolates under variable salinity, pH, and temperature.

Conditions		Strains			
		SNN087	SNN211	SNN289	SNN312
**Salinity**	0%	+	+	+	+
	4%	+	+	+	−
	7%	+	+	−	−
	10%	−	−	−	−
	13%	−	−	−	−
**pH**	5	+	+	+	+
	6	+	+	+	+
	7	+	+	+	+
	8	+	+	+	+
	9	+	+	+	+
**Temperature**	20°C	+	+	+	+
	30°C	+	+	+	+
	40°C	+	+	+	+
	50°C	−	−	−	−

+, growth observed; −, no growth observed.

### *Streptomyces* explorer cells triggered by *Saccharomyces cerevisiae*

**FIGURE 3:**
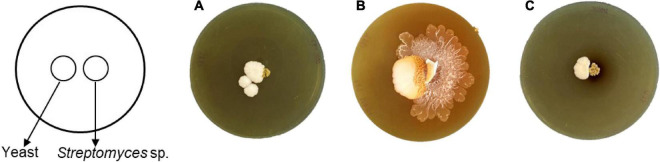
Exploratory behavior of *Streptomyces* isolates stimulated by *S. cerevisiae*. Colony morphology of the isolates grown beside *S. cerevisiae* after 17 days of incubation **(A)** SNN087, **(B)** SNN289, and **(C)** SNN312.

Interestingly, only strain SNN289 demonstrated a developmental life cycle trait similar to that of *S. venezuelae*, by consuming yeast cells in a co-culture setting prior to cell growth and rapid expansion to cover the surface of an agar plate (Figure 3). The three remaining strains did not exhibit exploratory growth.

### Mung bean growth promoted by selected *Streptomyces*

**FIGURE 4:**
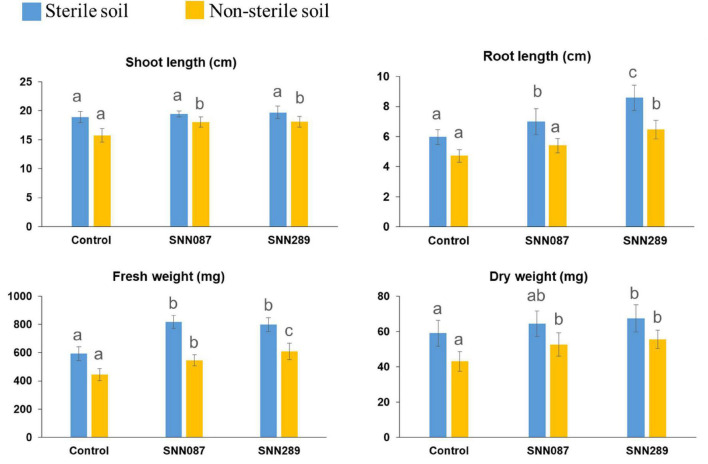
The effect of inoculated *Streptomyces* isolates on Mung bean (*Vigna radiata*) growth. The results are the means of ten plants ± SD from two independent biological replicates (20 plants in total). Different letters indicate significant differences at a *p* < 0.05 according to ANOVA analysis combined with the Tukey’s post-test.

**FIGURE 5:**
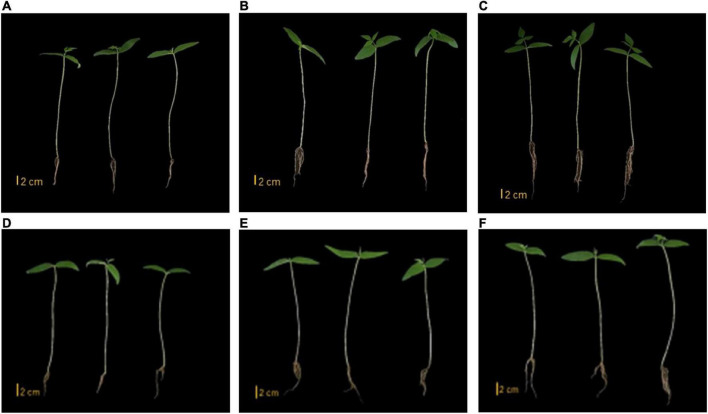
Mung bean growth in sterile soil. **(A)** Control **(B)** SNN087 treatment **(C)** SNN289 treatment. Mung bean growth in non-sterile soil. **(D)** Control **(E)** SNN087 treatment **(F)** SNN289 treatment.

We tested two isolates with significant plant-growth-promoting *in vitro* tested activities for their ability to enhance growth of mung bean seedlings in both sterile and non-sterile soil settings. Strains SNN087 and SNN289 exerted positive effects on mung bean growth in sterile soil, including root length and fresh and dry weights, while there was no statistically significant difference in shoot length between control and test strain-inoculated conditions (Figures 4, 5). Strain SNN289 dramatically enhanced root development, as reflected in significantly increased root length in the assay. In non-sterile soil, all growth-enhancing parameters were observed in both treatment groups (Figures 4, 5). Strikingly, strain SNN289 promoted improvement across all biometric parameters compared to untreated control soil. Compared to non-inoculated control, treatment with strain SNN087 significantly increased the plant biomass and shoot height but had no effect on root length. Overall, strain SNN289 promoted superior growth of mung bean plants compared to strain SNN087.

### The influence of strain SNN289 on microbial community structure

Since strain SNN289 exhibited such dramatic and broad effects on plant growth, we hypothesized that it may be affecting soil bacterial ecology or the composition of the soil microbial community. To test our hypothesis, we plotted the rarefaction curves of soil microbial samples subjected to metagenomic sequencing (Supplementary Figure 2) demonstrated the richness of the observed species and indicated that the sequencing depth was sufficient to completely capture the diversity of both bacterial and fungal communities. Read-based metagenome taxonomic profiling on Uclust from Qiime v.1.9.1 with the Zymo Research Database provided a comprehensive overview of microbial taxa within the sample. As shown in Supplementary Tables 1, 586 bacterial species and 198 fungal species were observed in the uninoculated control sample, while 815 bacterial species and 197 fungal species were observed in SNN289 treatment sample. We calculated the alpha diversity for each sample, which is a measurement of the microbial diversity of each sample. We also calculated the species richness (Shannon Index) and evenness (Gini/Simpson index) to give a measurement of community diversity. The greater the Shannon index value, the greater diversity of species present in the sampling population. For sample SNN289, we calculated a Shannon diversity index of 9.090 compared to the control soil 8.828 for bacteria species, indicating a greater abundance and richness of constituent bacterial species in the sample. In contrast, fungal diversity found in untreated soil was greater, a higher Shannon diversity index value (6.274) compared to 5.427 in the SNN289 treated sample.

**FIGURE 6:**
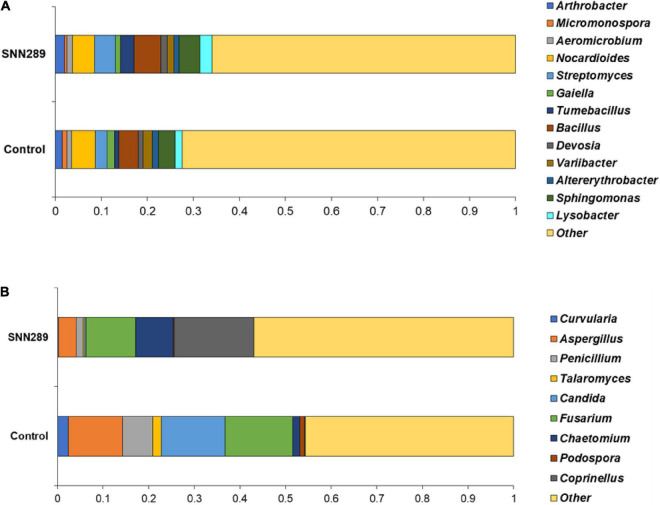
The relative abundance of dominant bacterial **(A)** and fungal **(B)** genera in non-sterile soils untreated and treated with *Streptomyces* sp. SNN289. The relative abundance was based on the proportional reads of DNA sequences classified at the genus level.

We investigated the relative abundance of classified bacterial genera more deeply in the SNN289 sample and found that treatment with *Streptomyces* sp. SNN289 led to more abundant bacterial and fungal biomass in the treated soil compared with untreated soil. The presence of each genus at greater than one percent of the population was considered dominant. In the bacterial community, 13 dominant genera were identified at the genus level, including *Pseudarthrobacter*, *Streptomyces*, *Tumebacillus*, *Bacillus*, *Devosia*, *Sphingomonas*, *Lysobacter*, *Aeromicrobium*, *Variibacter*, *Altererythrobacter*, and *Nocardioides* (Figure 6A). Bacteria from the genera *Pseudarthrobacter*, *Streptomyces*, *Tumebacillus*, *Bacillus*, *Devosia*, *Sphingomonas*, *Lysobacter*, and *Aeromicrobium* were enriched in the treatment soil relative to control soil, whereas the genera *Variibacter*, *Altererythrobacter*, and *Nocardioides* were declined (Figure 6A and Supplementary Figure 3).

In the fungal community, 9 identified fungal genera were dominant in the SNN289 sample, namely *Coprinellus*, *Chaetomium*, *Curvularia*, *Aspergillus*, *Penicillium*, *Talaromyces*, *Candida*, *Fusarium*, and *Podospora* (Figure 6B). The genera *Coprinellus* and *Chaetomium* were the most abundant. Notably, the genus *Coprinellus* accounted for a large portion of the treatment sample, whereas a small proportion (nearly zero) of this genus was present in control soil. In contrast, 7 fungal genera, including potential fungal pathogens *Fusarium*, were reduced in SNN289 compared to control (Figure 6B and Supplementary Figure 4).

### Genes contributing to plant-beneficial traits

Genome mining for genes encoding plant-beneficial properties offers the opportunity to scrutinize the genome of a PGPR strain. Based on this whole genome sequencing data, we estimated the genome sizes of *Streptomyces* sp. SNN087 and SNN289 to be 8.46 and 8.74 Mb, respectively, with GC content of 72.7 and 72.9%, respectively. We summarized the results of sequence assembly and genome annotation in Table 3. Genome annotation using the RAST server identified genes from 24 subsystem categories, with a total of 316 subsystems identified for strain SNN087, and 318 subsystems identified for strain SNN289 (Supplementary Figures 5, 6). This annotation revealed the presence of genes involved in several known growth promotion pathways (Table 3 and Supplementary Table 2), the most notable of which were genes responsible for siderophore production, auxin biosynthesis, nitrogen metabolism, and phosphate solubilization by an alkaline phosphatase. Genes involved in desferrioxamine E synthesis were found in both strains, while genes responsible for the siderophore, aerobactin, was only found in the SNN087 genome. Genes encoding proteins involved in nitrate/nitrite ammonification were identified in both strains, whereas genes encoding proteins responsible for catalyzing cyanate were identified only in the SNN289 genome.

**TABLE 3:** Comparison of genome features of strains SNN087 and SNN289 and their plant-beneficial genes in the genomes.

	SNN087	SNN289
Size (bp)	8,464,570	8,737,711
GC Content	72.7%	72.9%
N50	254797	231474
L50	12	10
Number of Contigs (with PEGs)	80	64
Number of Subsystems	316	318
Number of Coding Sequences	8023	8247
Number of RNAs	78	78
Iron acquisition		
- Siderophore assembly kit	+	+
- Siderophore desferrioxamine E	+	+
- Siderophore aerobactin	+	−
Plant hormone		
- Auxin biosynthesis	+	+
Ammonium production		
- Cyanate hydrolysis	−	+
- Nitrate/nitrite ammonification	+	+
Phosphate solubilization		
- Phosphate metabolism	+	+

+, present; −, absent.

### Biosynthetic gene clusters and predicted secondary metabolites

Since we observed obvious differences and some unexpected added novelty in the SNN289 genome, we next analyzed the genomes of both SNN087 and SNN289 computationally, for biosynthetic gene clusters (BGCs) and predicted secondary metabolite production which could be linked to the enhanced plant-growth phenotypes we observed. We used the AntiSMASH server which revealed the presence of 35 discrete biosynthetic clusters in strain SNN087 and 34 clusters in SNN289 (Table 4 and Supplementary Tables 3, 4), respectively. The most abundant BGCs identified in both strains were those encoding secondary metabolites including polyketides, non-ribosomal peptides, hybrids of polyketides and non-ribosomal peptides, terpenes, siderophores, ribosomally synthesized and post-translationally modified peptide products, and melanins. Comparing the two genomes, 18 clusters were identified in SNN087 and 12 clusters in SNN289 with greater than 20% similarity in potentially encoded compounds, such as spore pigment, melanin, hopene, alkylresorcinol, auricin, neothioviridamide, desferrioxamine B, geosmin, ectoine, coelichelin, carotenoid, albaflavenone, and actinomycin D. From 35 BGCs in the SNN087 genome, 9 BGCs demonstrated a low similarity (≤20%) to known clusters from other bacteria, and 8 BGCs were scored as likely to encode for possible novel compounds. Similarly, 13 clusters in the SNN289 genome were identified with a low (≤20%) similarity to known clusters, while 9 clusters were identified that potentially encode for new secondary metabolites.

**TABLE 4:** Predicted biosynthetic gene clusters encoding secondary metabolites.

		SNN087	SNN289
**Type**	PKS type I, II, III	4	6
	NRPS	5	4
	PKS-NRPS	5	3
	Terpene	5	4
	Siderophore	3	3
	RiPP	6	8
	PKS-RiPP	1	1
	Other	6	5
**Total**		**35**	**34**
**Similarity to known compounds**	>20%	18	12
	≤20%	9	13
	Unidentified	8	9
**Known compounds with 100% similarity**		geosmin coelichelin albaflavenone	alkylresorcinol auricin neothioviridamide desferrioxamine B

PKS, polyketide synthase; NRPS, non-ribosomal peptide synthetase; RiPP, ribosomally synthesized and post-translationally modified peptide product cluster.

## Discussion

The aim of this study was to undertake a systematic evaluation of *Streptomyces* strains for developing bioinoculants capable of promoting plant growth and controlling a phytopathogen that causes destructive diseases in economically important crops in Thailand. Currently, even though PGPR is an acceptable green strategy, the proportion of *Streptomyces*-based products that have been registered for commercial availability as bioinoculants is relatively low (Vurukonda et al., 2018). However, robust local plants with apparent resistance to the most devastating phytopathogen are thriving in several locales, suggesting that there may be additional relevant and highly potent *Streptomyces* species to be discovered and deployed.

Rhizosphere soil is the area in contact with the plant roots that is actively enriched with a complex mixture of nutrient sources provided by the plant to attract microorganisms (Bais et al., 2006). The assemblies of microorganisms found in the soil depend on the soil type, the host plant species, the host plant genotype, and on root system architecture (Ofek-Lalzar et al., 2014; Pérez-Jaramillo et al., 2017). Deep characterization of rhizosphere soil species diversity allows us to discover the bacteria engaged in the most effective mutualistic relationships with plants. A variety of natural compounds secreted by actinomycetes that live in the rhizosphere have been proven to aid in plant defense against pathogenic bacteria and fungi (Rungin et al., 2012; Suárez-Moreno et al., 2019). In addition, biogeographic studies on *Streptomyces* ecology have proposed that niches dominated by *Streptomyces* species with broad and extremely effective inhibitory phenotypes may act as competitive hot spots, implicating the *Streptomyces* as critical for sustaining plant health (Behie et al., 2017). As a result, all isolates in the current study were initially screened for antimicrobial property against an important plant pathogen using the co-culture technique.

Having identified *Streptomyces* isolates with strong inhibitory properties, we sought to characterize these strains more extensively, in order to better understand how they improve plant health. This required assaying the growth and metabolism (including secondary metabolite production), as well as the robustness of each strain to varying environmental growth conditions. We found several parameters in which our most promising strain, SNN289, excels in enhancing growth conditions. First, we observed that decreasing pH in the NBRIP medium was positively correlated with the amount of solubilized phosphate by each isolate, as organic acids produced by the isolates transform insoluble phosphate into available form. *Streptomyces* sp. SNN289 and SNN087 solubilized inorganic phosphate with relatively high amount as compared to an existing commercial strain, *Streptomyces* sp. CTM396 solubilizing 323 μg/ml (Farhat et al., 2015). Genome mining revealed that the genomes of the Streptomyces strains encode proteins linked to phosphate mineralization, such as an alkaline phosphatase (EC 3.1.3.1). Extracellular alkaline phosphatase catalyzes the transformation of a broad range of phosphate substrates into inorganic phosphate, including inorganic oligophosphates, polyphosphates, and organic phosphates (Moura et al., 2001).

The concentration of indole compounds produced by our test strains, as determined experimentally using Salkowski’s reagent, suggested that auxin biosynthesis in our strains is likely synthesized through tryptophan-dependent IAA biosynthesis. This finding would not be surprising, given a recent survey found that roughly 82.2% of bacterial genomes have the capacity to synthesize IAA from tryptophan or intermediates (Zhang et al., 2019). However, Salkowski’s reagent does not strictly distinguish IAA from IAA-like substances (Goswami et al., 2015). To address this constraint, an HPLC-based approach was devised for the more precise detection and quantification of IAA. This approach has standalone utility for the detection and quantification of IAA in microbiological samples without regard for other derivatives. For microbiological samples, the spectrophotometric method using Salkowski’s reagent yielded higher IAA values than the HPLC approach, which suggests that, indeed, other indole compounds may have reacted with the Salkowski’s reagent in our samples in addition to IAA. Nevertheless, the genomic presence of tryptophan biosynthesis genes, such as a tryptophan synthase alpha chain (EC 4.2.1.20) and a tryptophan synthase beta chain (EC 4.2.1.20), in our test strains predicts that IAA biosynthesis of the isolates is likely a tryptophan-dependent pathway (Raboni et al., 2009). Furthermore, the presence of a gene coding an aromatic-L-amino-acid decarboxylase (EC 4.1.1.28) suggested that IAA may also be synthesized through the tryptamine pathway, as that enzyme catalyzes the decarboxylation of L-tryptophan into tryptamine prior to converting into auxins (Yuwen et al., 2013; Subramaniam et al., 2020). Taken together, our results suggest that formation of indole compounds *via* tryptophan containing substrates or intermediates are a shared trait of our strains that support improved plant growth and support specific study of this ability in other rhizosphere strains.

While some *Streptomyces* spp. with nitrogen-fixing ability have been studied (Dahal et al., 2017), others can produce ammonia in peptone water broth by cleaving an amino functional group from protein backbone. This process converts nitrogen-containing organic compounds into available nitrogen for plants (Trivedi et al., 2020). Nitrate is reduced to nitrite by nitrate reductase, which then reduces nitrite to ammonia by a nitrite reductase [NAD(P)H] (EC 1.7.1.4) (Lam and Kuypers, 2011). This nitrate/nitrite ammonification is a critical step in the recycling of nitrogen. We report the identification of molecular machinery for hydrolysis of cyanate by a cyanate hydratase (EC 4.2.1.104) in strain SNN289, suggesting it may provide this strain the capacity to produce ammonium ion and carbon dioxide (Anderson et al., 1990).

We also investigated the ability of our strains to scavenge iron from the environment, which could provide valuable nutrients that promote plant growth. Desferrioxamines are the major siderophores for *Streptomyces* species (Yamanaka et al., 2005), and we identified desferrioxamine E in the genomes of both isolates using SEED viewer. Desferrioxamine B, which was identified in the genome of strain SNN289 with 100% similarity to other *Streptomyces* species, is more efficiently absorbed than desferrioxamine E (Müller and Raymond, 1984), which suggests an additional tool for iron assimilation in that strain. Having a diverse repertoire of iron-chelating agents may help these bacteria compete for the acquisition of the environmental ferric ion.

Exploratory growth behavior which induces the rapid outgrowth of vegetative hyphae is a special feature of *Streptomyces*, which can be induced by nutrient limiting conditions and is considered beneficial to the species in competing for limited resources. Exploratory growth can be induced as a result of a glucose deficiency (Jones et al., 2017), and low-iron environments can cause *Streptomyces venezuelae* to respond by secreting differently modified siderophores and upregulating genes involved in siderophore uptake. The capacity for inducing explorer cells is considered a benefit the bacteria, by allowing them to cover a greater area in natural habitats and outcompete other microorganisms. *Streptomyces* sp. SNN289 nicely and somewhat unexpectedly demonstrated this behavior, which probably allows SNN289 to dominate over other microbes in the rhizosphere.

This unexpected behavior led us to hypothesize that SNN289 may be a new species. *16S rRNA* sequences have been extensively used to classify and identify bacteria, with 98.65% similarity serving as the current cutoff for distinguishing species (Kim et al., 2012). Furthermore, high ANI (<95%) and high DDH values (Formula 2 < 70%) are typically reported for bacterial isolates from the same species and considered to indicate the limits of bacterial species boundaries (Konstantinidis and Tiedje, 2005). Our phylogenomic analysis placed strain SNN289 in a clade with *S. katrae*, while the ANI and dDDH values we calculated for this strain suggested that is likely not merely a strain variant of the species *S. katrae*. Therefore, SNN289 is potentially a new species closely related to *S. katrae* NRRL ISP-5550. *16S rRNA* gene analysis also indicated that strain SNN312 with 98.65% similarity to known species, had a high potential to be new species, but additional taxonomic studies are needed to confirm this result.

Since one of the long-term goals is to identify microbial species that are beneficial for biocontrol and plant growth promotion, we followed *in vitro* characterization of strains SNN087 and SNN289 with *in vivo* experiments to assess this potential, *in vivo*. To evaluate their *in vivo* capacity for growth promotion, these two strains were inoculated with sterile soil (to assess their maximum performance) and non-sterile soil (to assess the effect of inoculation on plant development in the presence of soil microbiota). We found that both strains could improve all the biometric parameters under sterile conditions, except dry weight in the SNN087 treatment. In non-sterile conditions, growth trends were similar to sterile conditions, except that root length and fresh weight in the SNN087 treatment were even more strikingly increased. Mung bean growth in SNN087 treatment under non-sterile conditions revealed a significant drop in plant growth measurements compared to sterile conditions, suggesting that strain SNN087 might be as fit in competition with others strains like SNN289 under more natural growth conditions. The reduced root length observed in the SNN087 treatment experiment could be explained by the negative effects of high IAA level, whereas increased root length occurred in the soil treated with SNN289 capable of producing IAA at lower concentrations. IAA at low concentrations has been reported to promote primary root elongation, shortening primary roots at high concentrations, and instead, favoring production of lateral roots, and increasing root hair development (Remans et al., 2008). Our results are consistent with a role for IAA in modulating plant growth. In addition to IAA, we hypothesize that available phosphate solubilized by bacteria also affected plant growth, since SNN289 solubilized inorganic phosphate significantly better than SNN087, *in vitro*. Nevertheless, our *in vivo* results will require further experiments assessing available nutrients in soil, interaction with plant host, plant transcriptomes, as well as metabolomic studies to better understand the ways in which our test strains increase the fitness of their plant hosts. Application of both SNN289 and SNN087 resulted in positive outcomes for host plants. Field experiments with various plants grown in natural conditions will yield further insights into the natural range and possible applicability of these strains to biocontrol and fertilization *in situ*.

Metagenomic analysis of treated soils revealed that the bacterial community in the bacterized soil was more abundant and diverse, as reflected in the Shannon indices of the samples. The genera *Bacillus*, *Sphingomonas*, *Lysobacter*, and *Arthrobacter* were dominant in the SNN289 treatment. These genera are well-characterized as beneficial microbes for plants. *Bacillus* spp. usually promote plant growth by various means similar to *Streptomyces* spp. (Kashyap et al., 2019). *Sphingomonas*, which has a known capacity for IAA production, phosphate solubilization, antifungal activity as well as hydrocarbon degradation such as polyaromatic hydrocarbon and phenolic compounds, may thus contribute to community success (Kim et al., 2020). The genus *Lysobacter* is regarded as an excellent source of new secondary metabolites that are beneficial for plants including β-lactams with substituted side chains, macrocyclic lactams, and macrocyclic peptide or depsipeptide antibiotics (Panthee et al., 2016), underscoring its relevance for plant disease control. *Arthrobacter* has also proven effective at suppressing wilt disease and enhancing plant growth, which are important features of biocontrol and plant-growth promotion (Tchuisseu et al., 2018; Zhang et al., 2020).

Perhaps most importantly, inoculation of plants *Streptomyces* sp. SNN289 shifted the structure of the fungal community, especially reducing soil-borne pathogens. The fungal genus *Fusarium* is abundant in soil, and the majority of its species are plant pathogens. The abundance *Fusarium* declined notably in SNN289 treated plants. Two beneficial fungal genera, *Coprinellus* and *Chaetomium*, both dramatically increased after introduction of SNN289. *Coprinellus curtus* GM-21 has shown disease-suppressive ability against *Rhizoctonia solani*, which causes bottom-rot disease in *Brassica campestris* (Chinese cabbage), and *Fusarium* sp., which causes rot disease in lettuces, tomatoes, and melons, by interfering hyphal formation (Nakasaki et al., 2007). *Chaetomium* spp. have also been reported to enhance plant growth parameters and induce plant immunity simultaneously (Song and Soytong, 2017). Thus, it seems likely that one of the most important plant growth-promoting effects of SNN289 is its effects on fungal community composition in rhizosphere soil.

Our results are similar to previous reports by Hu et al. (2020) and Wang et al. (2020), wherein inoculation of *Streptomyces* spp. in the soil increased representation of *Bacillus* spp., *Sphingomonas* spp., *Arthrobacter* spp., and *Pseudarthrobacter* spp. whereas *Fusarium* spp. were considerably decreased. It is hypothesized that the Gram-positive bacteria degraded fungal hyphae, converting them into nutrient sources (Ballhausen and de Boer, 2016). Our metagenomic analysis indicated that *Streptomyces* sp. SNN289 worked synergistically with other beneficial microbes, highlighting the importance of microbial interactions in microbial ecology and community composition, the cycling of nutrients in ecosystems, and reduction of plant disease incidence.

## Conclusion

We report the unusual discovery of a plant growth-promoting *Streptomyces* species with exploratory behavior. *Streptomyces* sp. SNN289 had several different direct impacts on plant growth *via* PGP characteristics such as phosphate solubilization, IAA, ammonia, and siderophore production. Our results suggest that it also has positive but indirect effects on plant production and pathogen control, through modification of the local soil microbiome. Therefore, we suggest that SNN289 represents a promising biocontrol and plant growth-promoting agent, which may contribute meaningfully to sustainable alternatives to agricultural production.

## Data availability statement

The data presented in this study are deposited in the GenBank repository, accession numbers ON759259–ON759262.

## Author contributions

NN and BI designed the study and performed the data analysis. WS advised on plant pot experiment. NN, WP, and BI wrote the manuscript. All authors contributed to the data interpretation.

## Footnotes

1
https://www.ezbiocloud.net
