# Supplementary material for: Plant growth-promoting properties of Streptomyces spp. isolates and their impact on mung bean plantlets’ rhizosphere microbiome
Source: Front Microbiol. 2022 Aug 25;13:967415. doi: 10.3389/fmicb.2022.967415 (PMC9453592; doi:10.3389/fmicb.2022.967415)
Supplement: Supplementary file 1 [file Data_Sheet_1.pdf]

Supporting Information

Plant growth-promoting properties of *Streptomyces* spp. isolates and their impact on mung bean plantlets' rhizosphere microbiome

Napawit Nonthakaew[1,2], Watanalai Panbangred[3], Wisuwat Songnuan[4] and Bungonsiri Intra[1,2*]

[1]*Department of Biotechnology, Faculty of Science, Mahidol University, Bangkok, Thailand*

[2]*Mahidol University-Osaka University Collaborative Research Center for Bioscience and Biotechnology, Bangkok, Thailand*

[3]*Research, Innovation and Partnerships Office – RIPO (Office of the President), King Mongkut's University of Technology Thonburi, Bangkok, Thailand*

[4]*Department of Plant Science, Faculty of Science, Mahidol University, Bangkok, Thailand*

*Correspondence: bungonsiri.int@mahidol.edu

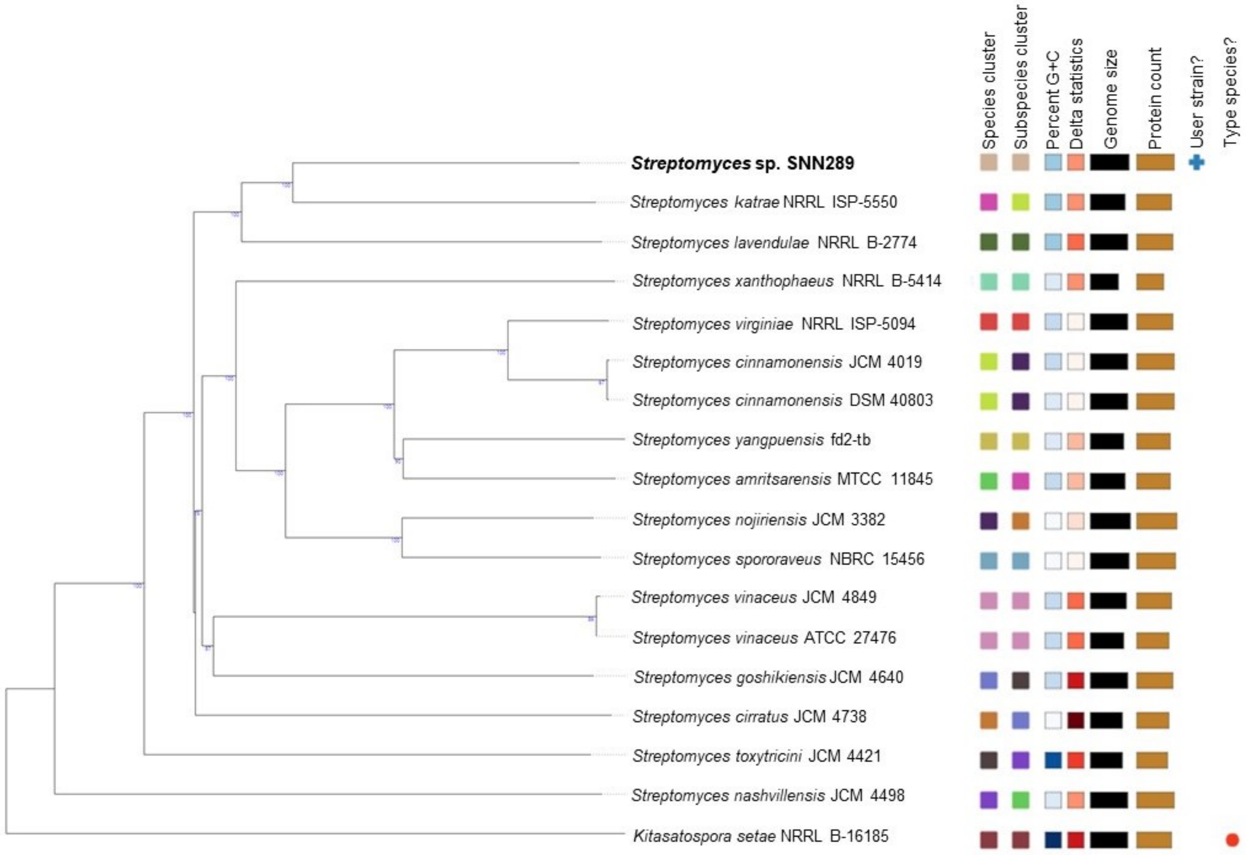

Supplementary Figure 1 Phylogenomic tree of *Streptomyces* sp. SNN289 and closely related strains of the genus *Streptomyces*. Tree inferred with FastME 2.1.6.1 (Lefort et al., 2015) from GBDP distances calculated from genome sequences. The branch lengths are scaled in terms of GBDP distance formula, d5. Numbers above each branch indicate GBDP pseudo-bootstrap support values > 60 % from 100 replicates, with an average branch support of 89.4 %. The tree was rooted at the midpoint (Farris, 1972).

(a)

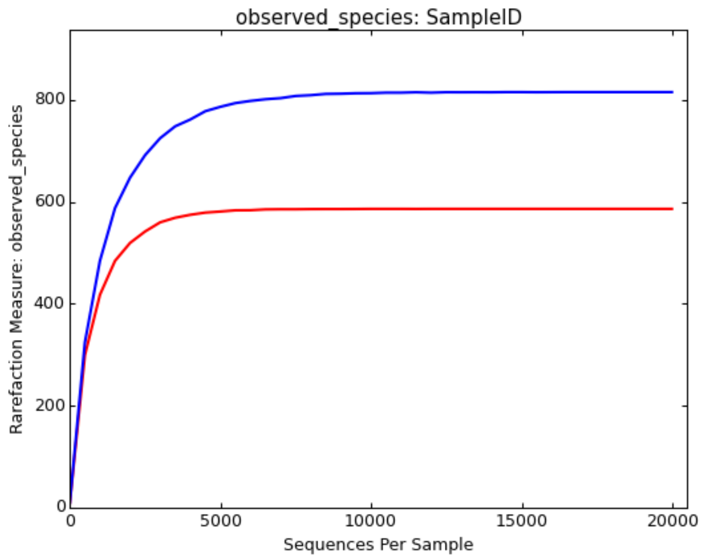

(b)

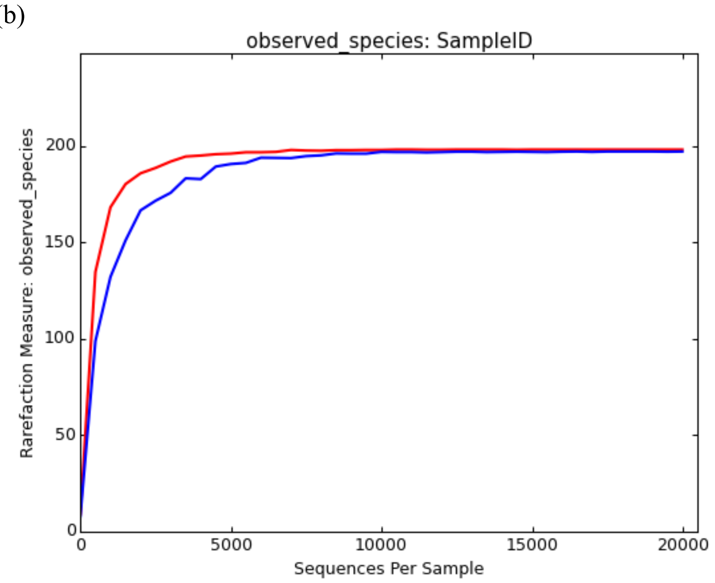

Supplementary Figure 2 The rarefraction curves of sequences obtained from samples for (a) bacteria and (b) fungi.

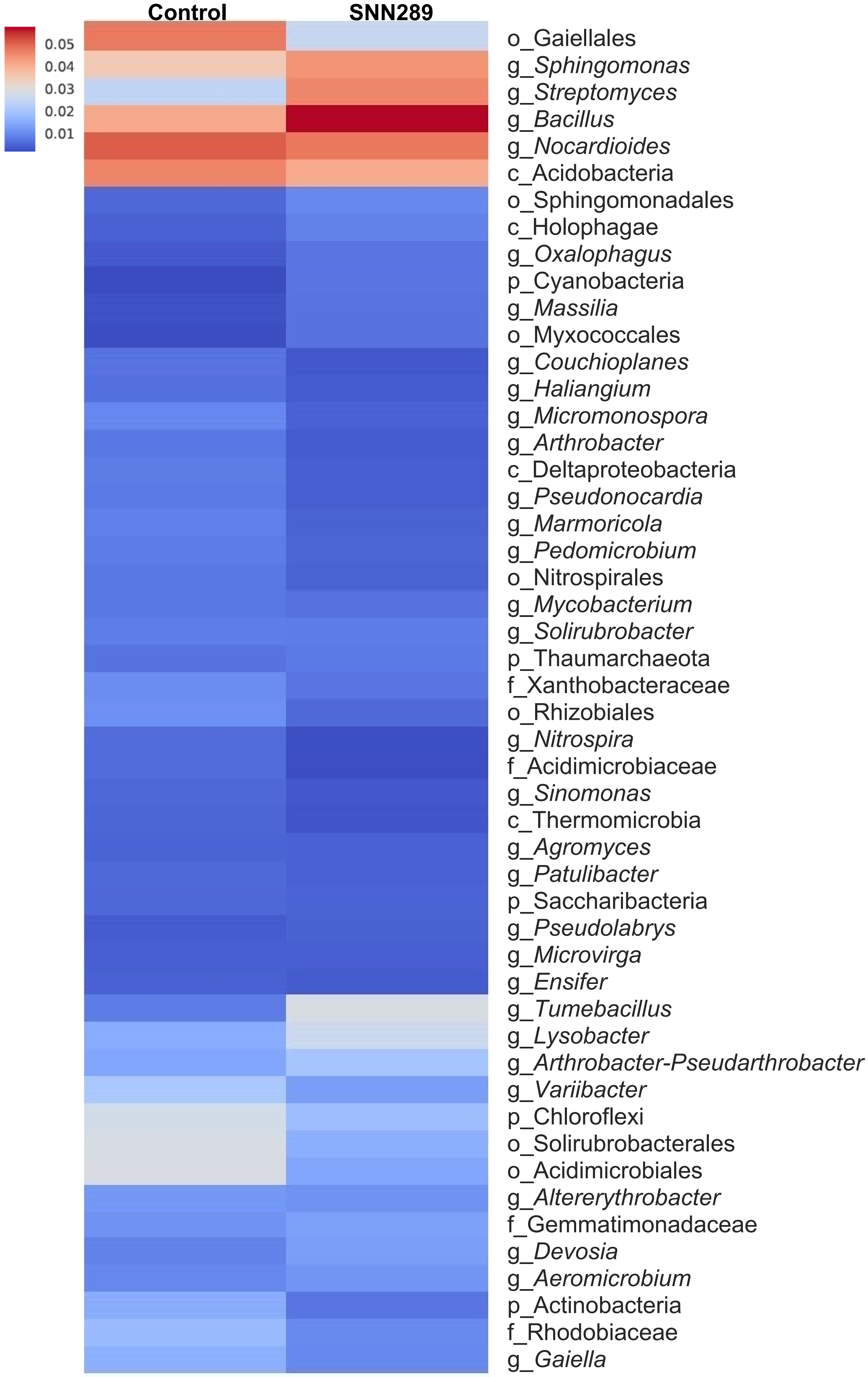

Supplementary Figure 3 Heatmap based on relative abundances of the top 50 bacterial species. The upper left corner depicts the color code for abundance. p, phylum; c, class; o, order; f, family; g, genus.

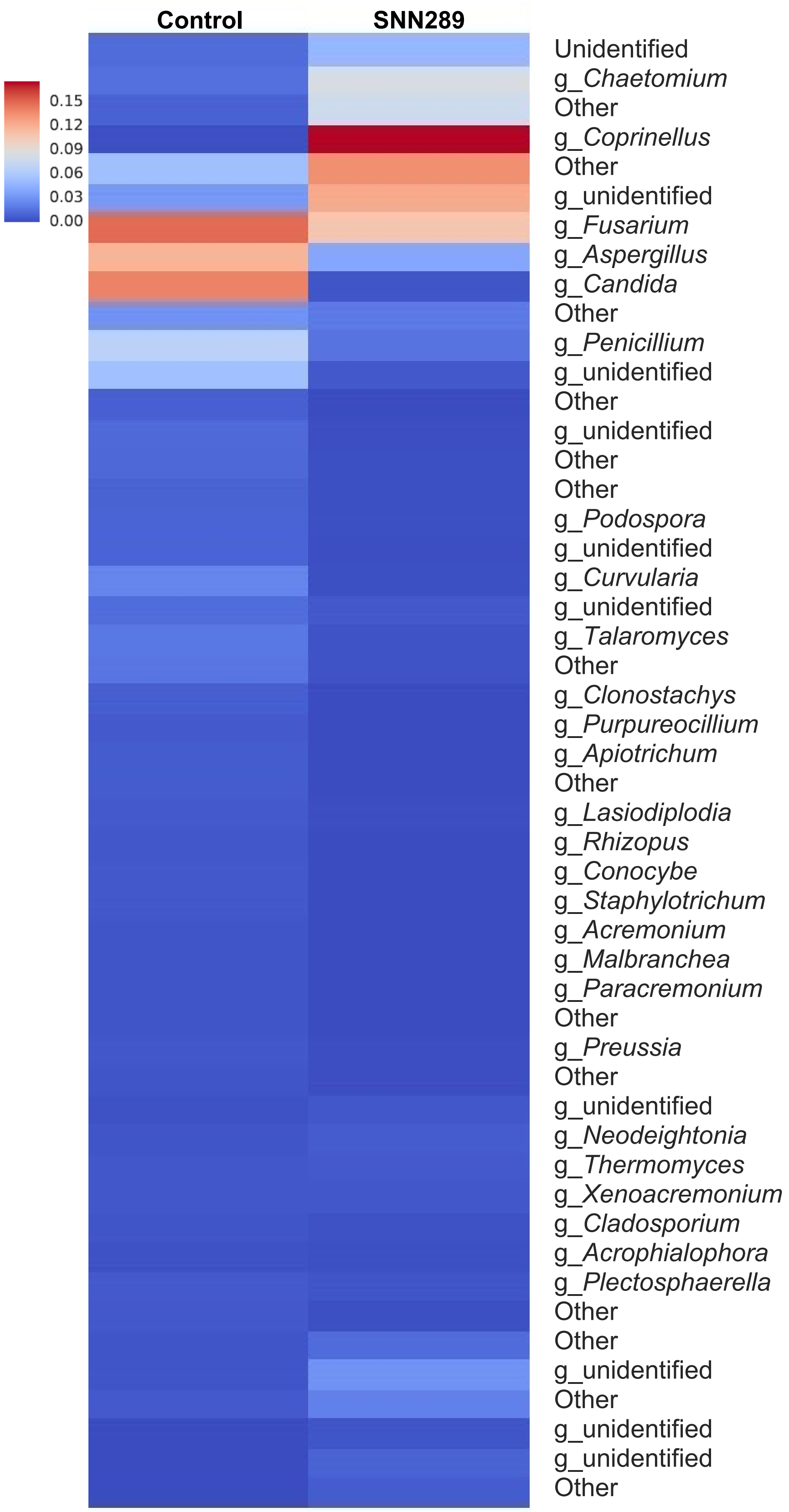

Supplementary Figure 4 Heatmap based on relative abundances of the top 50 fungal species. The upper left corner depicts the color code for abundance. g = genus.

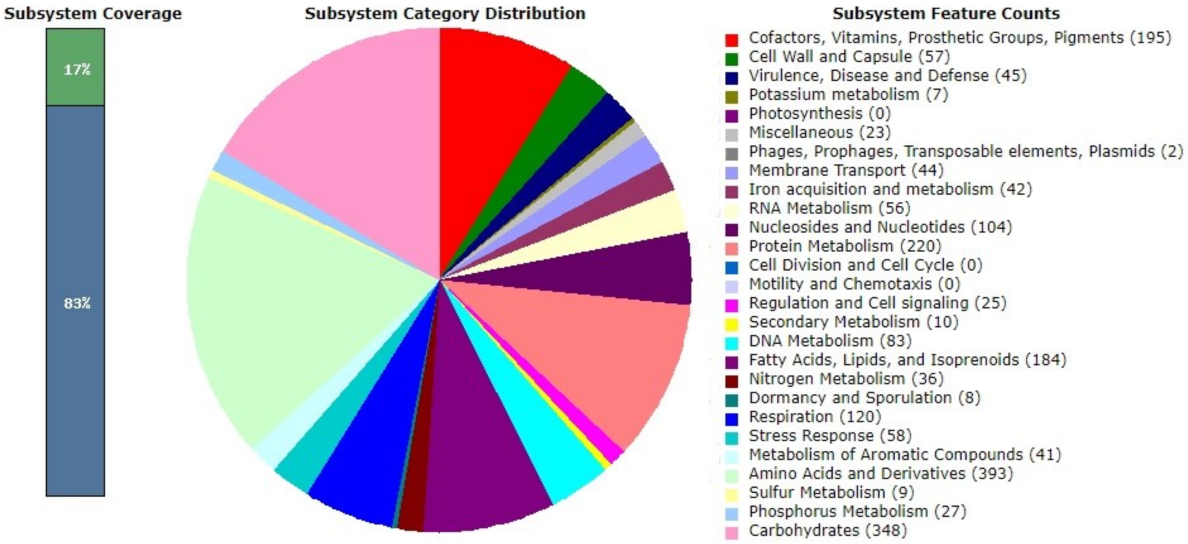

Supplementary Figure 5 Genome features of strain SNN087

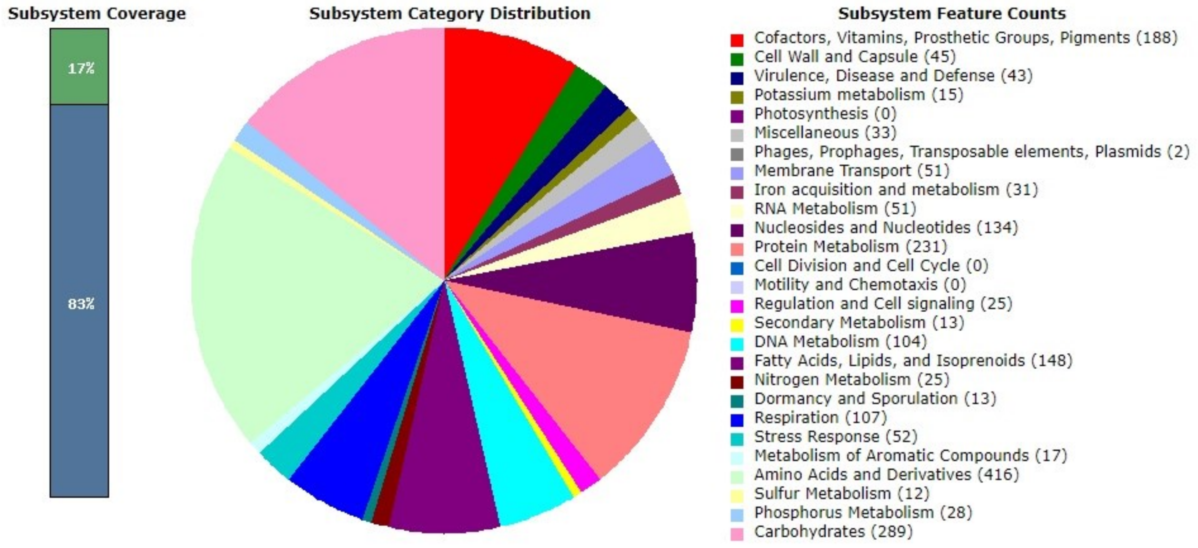

Supplementary Figure 6 Genome features of strain SNN289

Supplementary Table 1 Estimated richness and the Shannon diversity index of the rhizosphere soils

	Treatment	Community characteristics	
		Observed species	Shannon
16S	Control	586	8.828
	SNN289 treatment	815	9.090
ITS	Control	198	6.274
	SNN289 treatment	197	5.427

Supplementary Table 2 Proteins involved in PGP traits

<table>
<tr><th rowspan="2">PGP traits</th><th rowspan="2">Subsystem Features</th><th colspan="2">Protein linked to PGP traits</th></tr>
<tr><th>SNN087</th><th>SNN289</th></tr>
<tr><td>Iron acquisition</td><td>Siderophore assembly kit</td><td>1. Siderophore biosynthesis diaminobutyrate-2-oxoglutarate aminotransferase (EC 2.6.1.76)
2. Siderophore biosynthesis L-2,4-diaminobutyrate decarboxylase
3. Siderophore synthetase large component, acetyltransferase
4. Siderophore synthetase small component, acetyltransferase
5. Siderophore synthetase component, ligase
6. Siderophore biosynthesis protein, monooxygenase
7. Ferric hydroxamate ABC transporter (TC 3.A.1.14.3), permease component FhuB
8. Ferric hydroxamate ABC transporter (TC 3.A.1.14.3), ATP-binding protein FhuC
9. Ferric hydroxamate ABC transporter (TC 3.A.1.14.3), periplasmic substrate binding protein FhuD
10. Siderophore biosynthesis non-ribosomal peptide synthetase modules
11. Isochorismatase (EC 3.3.2.1) of siderophore biosynthesis
12. ABC-type Fe^{3+}-siderophore transport system, permease component
13. ABC-type Fe^{3+}-siderophore transport system, ATPase component
14. ABC-type Fe^{3+}-siderophore transport system, permease 2 component</td><td>1. Siderophore biosynthesis diaminobutyrate--2-oxoglutarate aminotransferase (EC 2.6.1.76)
2. Siderophore biosynthesis L-2,4-diaminobutyrate decarboxylase
3. Siderophore synthetase large component, acetyltransferase
4. Siderophore synthetase small component, acetyltransferase
5. Siderophore synthetase component, ligase
6. Siderophore biosynthesis protein, monooxygenase
7. Thioesterase in siderophore biosynthesis gene cluster
8. 2,3-dihydroxybenzoate-AMP ligase (EC 2.7.7.58)
9. ABC-type Fe^{3+}-siderophore transport system, permease component
10. ABC-type Fe^{3+}-siderophore transport system, ATPase component
11. ABC-type Fe^{3+}-siderophore transport system, permease 2 component</td></tr>
</table>

Supplementary Table 2 Proteins involved in PGP traits (cont.)

<table>
<tr><th rowspan="2">PGP traits</th><th rowspan="2">Subsystem Features</th><th colspan="2">Protein linked to PGP traits</th></tr>
<tr><th>SNN087</th><th>SNN289</th></tr>
<tr><td rowspan="2">Iron acquisition</td><td>Siderophore Desferrioxamine E</td><td colspan="2">1. Putative Desferrioxamine E transporter
2. Desferrioxamine E biosynthesis protein DesA
3. Desferrioxamine E biosynthesis protein DesB
4. Desferrioxamine E biosynthesis protein DesC
5. Desferrioxamine E biosynthesis protein DesD</td></tr>
<tr><td>Siderophore Aerobactin</td><td>1. Ferric hydroxamate ABC transporter (TC 3.A.1.14.3), periplasmic substrate binding protein FhuD
2. Ferric hydroxamate ABC transporter (TC 3.A.1.14.3), ATP-binding protein FhuC
3. Ferric hydroxamate ABC transporter (TC 3.A.1.14.3), permease component FhuB</td><td>-</td></tr>
<tr><td>Plant hormone</td><td>Auxin biosynthesis</td><td colspan="2">1. Anthranilate phosphoribosyltransferase (EC 2.4.2.18)
2. Phosphoribosylanthranilate isomerase (EC 5.3.1.24)
3. Tryptophan synthase alpha chain (EC 4.2.1.20)
4. Tryptophan synthase beta chain (EC 4.2.1.20)
5. Aromatic-L-amino-acid decarboxylase (EC 4.1.1.28)</td></tr>
<tr><td rowspan="2">Nitrogen metabolism</td><td>Cyanate hydrolysis</td><td>-</td><td>1. Cyanate hydratase (EC 4.2.1.104)</td></tr>
<tr><td>Nitrate and nitrite ammonification</td><td colspan="2">1. Nitrite reductase [NAD(P)H] large subunit (EC 1.7.1.4)
2. Nitrite reductase [NAD(P)H] small subunit (EC 1.7.1.4)
3. Nitrate/nitrite transporter</td></tr>
<tr><td>Phosphorus solubilization</td><td>Phosphate metabolism</td><td colspan="2">1. Alkaline phosphatase (EC 3.1.3.1)</td></tr>
</table>

Supplementary Table 3 Predicted secondary metabolites from SNN087 genome

Cluster	Type	From	To	Most similar known cluster		Similarity
1	siderophore	202,374	214,143	desferrioxamine	Other	83%
2	melanin	295,349	305,822	istamycin	Saccharide	7%
3	siderophore	61,689	73,208	-	-	-
4	RiPP-like	277,139	288,440	-	-	-
5	terpene	309,013	331,190	geosmin	Terpene	100%
6	siderophore	443,460	456,619	-	-	-
7	ectoine	1	5,782	ectoine	Other	75%
8	NAPAA	318,109	351,969	-	-	-
9	lanthipeptide-class-iii	188,294	211,026	labyrinthopeptin A2 / labyrinthopeptin A1 / labyrinthopeptin A3	RiPP:Lanthipeptide	60%
10	terpene, melanin	44,714	65,766	melanin	Other	57%
11	T1PKS, resorcinol, NRPS	77,890	200,324	coelichelin	NRP	100%
12	NRPS, T1PKS	243,097	295,210	herboxidiene	Polyketide	10%
13	T1PKS, hglE-KS	32,056	83,652	miharamycin A / miharamycin B	Polyketide	7%
14	redox-cofactor	92,560	114,828	lankacidin C	NRP + Polyketide	13%
15	RiPP-like	2,806	13,093	-	-	-
16	RiPP-like	90,945	101,160	informatipeptin	RiPP:Lanthipeptide	57%
17	indole	166,802	187,959	5-isoprenylindole-3-carboxylate β-D-glycosyl ester	Other	23%
18	T2PKS	1	63,127	spore pigment	Polyketide	83%
19	NRPS	68,926	113,248	diisonitrile antibiotic SF2768	NRP	66%
20	terpene	147,854	173,376	carotenoid	Terpene	63%
21	β-lactam, NRPS, NRPS-like, T1PKS	43,535	187,078	thienamycin	Other:Non-NRP beta-lactam	33%
22	terpene	59,677	80,774	albaflavenone	Terpene	100%
23	T1PKS	25,760	72,434	clifednamide A	NRP + Polyketide	30%
24	T3PKS, thiopeptide	3,080	85,280	granaticin	Polyketide:Type II	13%

Supplementary Table 3 Predicted secondary metabolites from SNN087 genome (cont.)

Cluster	Type	From	To	Most similar known cluster		Similarity
25	T1PKS, NRPS	97,899	132,592	s56-p1	NRP	11%
26	terpene	93,821	112,362	hopene	Terpene	53%
27	terpene	93,821	112,362	hopene	Terpene	53%
28	NRPS, transAT-PKS	18,845	83,784	oxalomycin B	NRP + Polyketide	12%
29	other	1	28,812	actinomycin D	NRP	71%
30	NRPS	29,406	52,692	actinomycin D	NRP	28%
31	NRPS	8,430	34,654	borrelidin	Polyketide: Modular type I	9%
32	thioamitides	1	14,624	-	-	-
33	T1PKS	1	8,193	-	-	-
34	NRPS	1	5,279	friulimicin A / friulimicin B / friulimicin C / friulimicin D	NRP	9%
35	NRPS	1	5,055	-	-	-

CDPS, tRNA-dependent cyclodipeptide synthases; hglE-KS, heterocyst glycolipid synthase-like PKS; NAPAA, non-alpha poly-amino acids like ε-Polylysine; NRPS, Non-ribosomal peptide synthetase cluster; NRPS-like, NRPS-like fragment; RiPP-like, Other unspecified ribosomally synthesized and post-translationally modified peptide product (RiPP) cluster; T1PKS, Type I PKS (Polyketide synthase); T2PKS, Type II PKS (Polyketide synthase); T3PKS, Type III PKS (Polyketide synthase).

Supplementary Table 4 Predicted secondary metabolites from SNN289 genome

Cluster	Type	From	To	Most similar known cluster		Similarity
1	T2PKS	173,984	246,505	spore pigment	Polyketide	66%
2	CDPS	560,865	581,575	-	-	-
3	melanin	70,512	79,817	melanin	Other	28%
4	melanin	91,063	101,428	istamycin	Saccharide	4%
5	terpene	105,531	125,360	monensin	Polyketide	5%
6	terpene	164,851	186,050	ebelactone	Polyketide	5%
7	terpene	327,178	345,030	-	-	-
8	T1PKS, hglE-KS	528,768	600,989	-	-	-
9	terpene	628,949	655,892	hopene	Terpene	61%
10	T3PKS	1	32,858	alkylresorcinol	Polyketide	100%
11	LAP,thiopeptide,NAPAA	160,651	208,686	lactazole	RiPP:Thiopeptide	33%
12	T2PKS, butyrolactone, NRPS-like, T1PKS	3	151,276	auricin	Polyketide:Type II + Saccharide:Hybrid /tailoring	100%
13	NRPS, other, T1PKS, melanin	170,927	342,519	aurantimycin A	NRP + Polyketide	57%
14	NRPS	89,931	142,892	nogalamycin	Polyketide	30%
15	lanthipeptide-class-i	166,529	192,982	-	-	-
16	NRPS, NRPS-like	1	34,151	-	-	-
17	lanthipeptide class-v, thioamitides	223,546	265,545	neothioviridamide	RiPP	100%
18	siderophore	87,480	99,264	desferrioxamine B	Other	100%
19	thiopeptide	65,190	89,760	funisamine	Polyketide	5%
20	T3PKS	114,833	155,987	herboxidiene	Polyketide	2%
21	nucleoside	13,138	33,515	auroramycin	Polyketide	5%
22	ladderane, NRPS	81,028	139,844	CDA1b / CDA2a / CDA2b / CDA3a / CDA3b / CDA4a / CDA4b	NRP:Ca+-dependent lipopeptide	27%
23	thiopeptide, LAP	148,348	179,205	lactazole	RiPP:Thiopeptide	44%
24	CDPS	21,869	42,624	BD-12	NRP	17%
25	siderophore	65,250	79,867	ficellomycin	NRP	3%
26	NRPS-like, T1PKS	114,532	145,803	auroramycin	Polyketide	19%
27	RiPP-like	13,231	20,770	-	-	-
28	RiPP-like	51,850	63,142	-	-	-

Supplementary Table 4 Predicted secondary metabolites from SNN289 genome (cont.)

Region	Type	From	To	Most similar known cluster		Similarity
29	lanthipeptide class-ii, lanthipeptide class-iii, lassopeptide, T1PKS	33,852	99,593	kanamycin	Saccharide	7%
30	thioamitides	1	21,135	-	-	-
31	NRPS	1	31,207	steffimycin D	Polyketide:Type II + Saccharide:Hybrid /tailoring	16%
32	T1PKS	1	28,427	tautomycetin	Polyketide: Modular type I	9%
33	siderophore	6,543	16,426	-	-	-
34	T1PKS	1	10,648	chlorothricin / deschlorothricin	Polyketide: Modular type I + Polyketide: Iterative type I + Saccharide: Oligosaccharide	11%

CDPS, tRNA-dependent cyclodipeptide synthases; hglE-KS, heterocyst glycolipid synthase-like PKS; LAP, Linear azol(in)e-containing peptides; NAPAA, non-alpha poly-amino acids like ε-Polylysine; NRPS, Non-ribosomal peptide synthetase cluster; NRPS-like, NRPS-like fragment; RiPP-like, Other unspecified ribosomally-synthesized and post-translationally modified peptide product (RiPP) cluster; T1PKS, Type I PKS (Polyketide synthase); T2PKS, Type II PKS (Polyketide synthase); T3PKS, Type III PKS (Polyketide synthase).
